# Using electroretinograms and multi-model inference to identify spectral classes of photoreceptors and relative opsin expression levels

**DOI:** 10.7717/peerj.3595

**Published:** 2017-07-21

**Authors:** Nicolas Lessios

**Affiliations:** 1 School of Life Sciences, Arizona State University, Tempe, AZ, USA; 2 Current affiliation: Department of Neuroscience, University of Arizona, Tucson, AZ, USA

**Keywords:** Color vision, Electrophysiology, Visual system, Spectral sensitivity, Photoreceptor, Opsin expression, Visual pigments

## Abstract

Understanding how individual photoreceptor cells factor in the spectral sensitivity of a visual system is essential to explain how they contribute to the visual ecology of the animal in question. Existing methods that model the absorption of visual pigments use templates which correspond closely to data from thin cross-sections of photoreceptor cells. However, few modeling approaches use a single framework to incorporate physical parameters of real photoreceptors, which can be fused, and can form vertical tiers. Akaike’s information criterion (AIC_c_) was used here to select absorptance models of multiple classes of photoreceptor cells that maximize information, given visual system spectral sensitivity data obtained using extracellular electroretinograms and structural parameters obtained by histological methods. This framework was first used to select among alternative hypotheses of photoreceptor number. It identified spectral classes from a range of dark-adapted visual systems which have between one and four spectral photoreceptor classes. These were the velvet worm, *Principapillatus hitoyensis*, the branchiopod water flea, *Daphnia magna*, normal humans, and humans with enhanced S-cone syndrome, a condition in which S-cone frequency is increased due to mutations in a transcription factor that controls photoreceptor expression. Data from the Asian swallowtail, *Papilio xuthus*, which has at least five main spectral photoreceptor classes in its compound eyes, were included to illustrate potential effects of model over-simplification on multi-model inference. The multi-model framework was then used with parameters of spectral photoreceptor classes and the structural photoreceptor array kept constant. The goal was to map relative opsin expression to visual pigment concentration. It identified relative opsin expression differences for two populations of the bluefin killifish, *Lucania goodei*. The modeling approach presented here will be useful in selecting the most likely alternative hypotheses of opsin-based spectral photoreceptor classes, using relative opsin expression and extracellular electroretinography.

## Introduction

Animals possess a diversity of opsin proteins, one of the main genetic components underlying spectral photoreceptor classes (Porter et al., 2012). It is now possible to identify functional amino acid sequence sites of opsin proteins that determine the spectral sensitivity of photoreceptors (Arendt et al., 2004; Porter et al., 2007). The number and wavelength sensitivity of spectral photoreceptor classes an organism possesses is needed to understand whether it can discriminate natural spectra (i.e., has some form of color vision), and also to understand the mechanistic context of visually guided behavior (Kelber & Osorio, 2010). Spectral classes of photoreceptors are generally identified using a combination of extracellular and intracellular electroretinographic (ERG) techniques (Arikawa, Inokuma & Eguchi, 1987). Extracellular recordings detect a summed contribution of multiple classes of photoreceptors, including relatively rare classes that are difficult to identify using intracellular techniques. It is possible to isolate spectral photoreceptor classes using chromatic adaptation, where light of a restricted waveband is used to light-adapt single photoreceptor classes and the resulting effects on spectral sensitivity are observed in extracellular recordings. However, because visual pigments are all natively sensitive to short wavelengths (Bowmaker, 1999), this procedure is most applicable to long wavelength receptors in organisms that possess up to three spectral photoreceptor classes (Goldsmith, 1986). Intracellular techniques are the most accurate for verifying the existence of spectral classes; but they can be further supported by modeling approaches which incorporate physical parameters obtained from histological techniques (Stavenga & Arikawa, 2011).

I have developed a framework of multi-model selection using overall spectral sensitivities of the visual system. The goals of this framework were to:

- Identify the most likely number of opsin-based spectral photoreceptor classes of visual systems from extracellular ERGs, and from known parameters of the photoreceptor array.
- Establish whether differences between individuals in structural photoreceptor parameters affect identification of the same underlying number of opsin-based spectral photoreceptor classes found in A.
- Map relative opsin expression levels to relative visual pigment concentrations when structural parameters and opsin identities of the photoreceptor array are known.

The framework used here employs Akaike’s information criterion (AIC_c_) to select among competing alternative hypotheses (Akaike, 1974). AIC is an objective measure that imposes a realistic penalty for over-parameterization (Burnham & Anderson, 2002). For goals A) and B) the alternative hypotheses are the number and relative area in cross-section, or frequency, of spectral photoreceptor classes. For goal C), the alternative hypotheses are the number of opsins which differ in relative expression level. Others have used multi-model selection to identify the number of photoreceptors in the eyes of oceanic fish, using the relative contributions of photoreceptor classes in cross-section to absorbance (Horodysky et al., 2008, 2010). Existing models of absorptance, which use parameters of real photoreceptors (Snyder, Menzel & Laughlin, 1973), are developed here to incorporate parameters of multiple tiers, or to model absorptive layers affecting the spectral sensitivity of underlying photoreceptors.

## Materials and Methods

### Visual modeling of photoreceptor absorptance

The fused photoreceptor array per unit length was modeled as

(1) $${{\rm{\xi }}_j}({\rm{\lambda }}) = \sum {{{\rm{\alpha }}_i}({\rm{\lambda }}){{{A_i}} \over A}k}, $$

where α_*i*_ is the normalized absorption spectrum of each rhodopsin visual pigment, *A_i_/A* is the relative area or frequency in cross-section of each photoreceptor *i*, and *k* is the peak absorption coefficient. Values used for *k* for invertebrates (0.008 μm^−1^) were established by Bruno, Barnes & Goldsmith (1977) and are typical for crustaceans and insects (Cronin et al., 2014). Values used for *k* for humans (0.015 μm^−1^) are typical for vertebrates (Wyszecki & Stiles, 1982). Absorptance of a tiered photoreceptor array, composed of *j* tiers was calculated as follows:

(2) $$S({\rm{\lambda }}) = \sum {\left({{T_{(j-1)}}\left({1-{e^{-{{\rm{\xi }}_j}({\rm{\lambda }}){l_j}}}} \right)} \right)} $$

where *T_j_*_−1_ is the transmittance through all preceding vertical tiers (*T*_0_ = 1.0 for the first tier). Normalized absorbance templates developed by Stavenga, Smits & Hoenders (1993), referred to here as SSH, and by Govardovskii et al. (2000), referred to here as GFKRD, were used for visual pigment absorption spectra α_*i*_, each of which has a wavelength of peak absorbance λ_max_. Normalized absorption templates have two primary components, an alpha band with a wavelength of peak absorbance that is determined by the interaction between the chromophore and the opsin protein, and a beta band which absorbs in the UV, and is mainly determined by the chromophore itself (Bowmaker, 1999). Effects of including both alpha and beta bands were assessed in a preliminary analysis of a global model, then only alpha bands were considered (see AIC_c_ procedure). *S*(λ) was normalized to 1 as in Stavenga & Arikawa (2011).

### Example selection

I used organisms which have between one and five classes of spectral photoreceptors to examine capabilities and limitations of the described framework. Four organisms were used to address goals A) and B), and spectral sensitivities from dark-adapted eyes were used to minimize effects of variation among individuals of changing visual pigment concentration, pigment migration, or varying levels of metarhodopsin (Stavenga, 2010). The fifth organism was used to address goal C) to map differences in visual pigment concentrations to relative opsin expression level for two populations of the same species.

**Figure 1:**
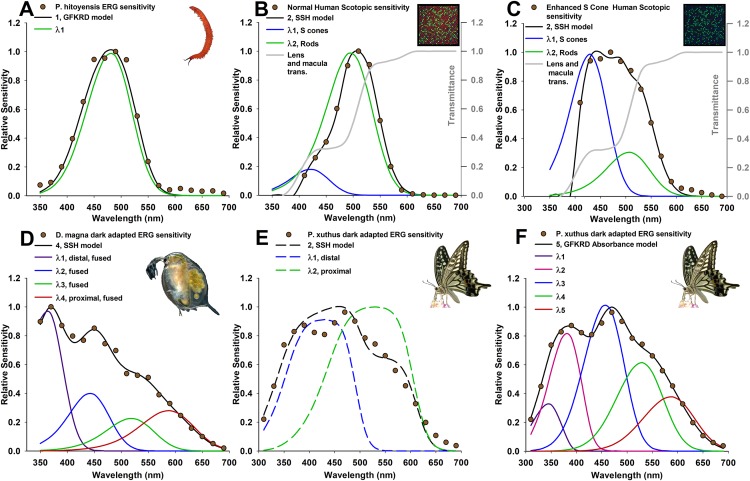
Photoreceptor absorptance models (curves) based on known photoreceptor lengths and vertical tiering, fit to relative spectral sensitivity data extracted from published sources (data points). Models were selected using Akaike’s information criterion corrected for small sample sizes (AIC_c_) with the best three models shown in Tables 1 and 2, and all models in Tables S1 and S2. (A) Velvet worm *Principapillatus hitoyensis* sensitivity, known to be represented by a single spectral opsin class expressed in its photoreceptors (Beckmann et al., 2015). (B and C) Normal and enhanced S-cone human scotopic sensitivities, known for normal humans to be represented by S-class cone and rod photoreceptor sensitivities, and with a higher frequency of S cones in patients that have enhanced S-cone syndrome (Jacobson et al., 1990; Hood et al., 1995; Haider et al., 2000). Absorptance models for humans are corrected for transmittance through the lens and a distal macula layer which protects the retina, but which does not contribute to spectral sensitivity (gray lines) (Wyszecki & Stiles, 2000). (D) *Daphnia magna* sensitivity, known to be represented by four spectral photoreceptor classes with a distal UV receptor (Smith & Macagno, 1990). (E and F) *Papilio xuthus* sensitivity, averaged from extracellular recordings from multiple positions in the compound eye, known to be represented by at least five main spectral photoreceptor classes (Arikawa, Inokuma & Eguchi, 1987). (E) Absorptance models (dashed lines) illustrate poor results with this technique because of model over-simplification explained in text. (F) Absorbance (given by Eq. (1)) at a cross-section approximately two-thirds from the distal tip of the rhabdom of an ommatidium selects five spectral photoreceptor classes, with deviations of each spectral class explained further in the text due to specialized filtering pigments.

1. The onycophoran velvet worm, *Principapillatus hitoyensis* (Fig. 1A) expresses a single spectral opsin class in its photoreceptors (Beckmann et al., 2015).
2. *Homo sapiens* possesses one rod and three cone (S, M, and L) photoreceptor classes. Normal human scotopic sensitivity (Fig. 1B) is represented by S-class cone and rod photoreceptor sensitivities (Bowmaker & Dartnall, 1980; Wyszecki & Stiles, 2000). In contrast, scotopic sensitivity of patients with enhanced S-cone syndrome (Fig. 1C) is a condition in which S-cone frequency is increased due to mutations in a transcription factor that controls photoreceptor expression (Haider et al., 2000). Human absorptance models are corrected here for transmittance through the lens and a distal macula tier protecting the retina that affects spectral sensitivity (Wyszecki & Stiles, 1982).
3. The branchiopod crustacean water flea, *Daphnia magna* (Fig. 1D) possesses four spectral photoreceptor classes (Smith & Macagno, 1990).
4. The swallowtail butterfly, *Papilio xuthus* (Figs. 1E and 1F) possesses at least five main spectral classes of photoreceptor type (Arikawa, Inokuma & Eguchi, 1987), in several classes of ommatidia with specialized filtering pigments (Stavenga & Arikawa, 2011).
5. The bluefin killifish, *Lucania goodei*, possesses five cone photoreceptor classes based on known opsins (SWS1, SWS2B, SWS2A, RH2-1, and LWS). Separate populations of this species have been shown to regulate opsin expression depending on their photic environments (Fuller et al., 2004). Killifish absorptance models are corrected here for transmittance through a tier of distal ellipsosomes associated with cone classes found in the related killifish *Fundulus heteroclitus* (Flamarique & Harosi, 2000), and through the lens of the Nile tilapia *Oreochromis niloticus* (Lisney, Studd & Hawryshyn, 2010). The relative frequency of the cones cone classes that express SWS2B, RH2-1, and LWS were corrected to take into account that they are double cones.

### Data extraction, binning, and averaging from multiple recording locations

Published spectral sensitivity data were extracted using GetData v.2.26 (Fedorov, 2013) from Arikawa, Inokuma & Eguchi (1987), Smith & Macagno (1990), Jacobson et al. (1990), Fuller et al. (2003) and Beckmann et al. (2015). Where needed, units were converted from log sensitivity to relative sensitivity. Preliminary analysis indicated that 20 and 10 nm wavelength intervals provided identical results. Binning was therefore carried out at 20 nm intervals for all sensitivity data. Sensitivity ranges were 410–690 nm for humans, 350–690 nm for *Principapillatus hitoyensis* and *D. magna*, and 310–690 nm for *P. xuthus*. For *P. xuthus* (Arikawa, Inokuma & Eguchi, 1987) had recorded extracellularly from multiple regions of the compound eye (dorsal, medial, and ventral). Binned sensitivities from each region were therefore averaged to provide a single relative spectral sensitivity (Figs. 1E and 1F).

### Incorporating known photoreceptor lengths *l_j_* in Eq. (2)

Photoreceptor lengths were estimated or taken from published sources: *Principapillatus hitoyensis* (100 μm) (Beckmann et al., 2015); *H. sapiens* parafovea (22.5 μm) (Bowmaker & Dartnall, 1980; Cronin et al., 2014); *D. magna* (12.0 μm) (Smith & Macagno, 1990); *Papilio xuthus* (500 μm) (Arikawa & Stavenga, 1997); and *L. goodei* (18 μm) (Moldstad, 2008). The fused cross-sectional and tiered three-dimensional photoreceptor array is known for *D. magna* and for *P. xuthus*: as in many insects and crustaceans (Kelber & Henze, 2013), the shortest wavelength receptor of both species becomes axon-like partway through the optical unit. Models considered here for *D. magna* and *P. xuthus* which have more than one spectral class of photoreceptor incorporate this structure in Eq. (2), and in the optimization procedure. The shortest wavelength receptor of *D. magna* ommatidia forms a fused structure in the distal (upper) half of the optical unit (6.0 μm), with a short-wavelength receptor replaced by a longer-wavelength sensitive receptor in the proximal (lower) half of the optical unit (6.0 μm). The distal two-thirds of the optical unit (333 μm) of *P. xuthus* ommatidia is modeled as a single optical unit, replaced by a long wavelength receptor in the proximal portion (167 μm).

### Parameter estimates, maximum likelihood estimation, optimization, and AIC_c_ procedure

The maximum likelihood estimate (MLE) of each model was calculated according to Burnham & Anderson (2002)

(3) $$ log (L(\underline{\hat \Theta} )) =  - {1 \over 2}log ({\underline{{\hat \sigma}^2}}) - {n \over 2}log (2\pi ) - {n \over 2},$$

where the MLE for ${\hat \sigma}^{\rm{2}}$ is ${{{{RSS}}} \over n}$, and *RSS* is the residual sum of squares for a given model. Optimization of model parameters λ_max_, and *A_i_*/*A* for goals A) and B), then *k* for goal C) were carried out using custom scripts, and the optimization toolbox in MATLAB. A linear constraint was used for *D. magna* and *P. xuthus* during optimization to maintain λ_max1_ as the shortest wavelength receptor in the first tier (λ_max *i*_ < λ_max *i*+1_). The absorption coefficients for *L. goodei* were constrained to a value greater than 0.001/μm and less than 1.000/μm.

I used AIC_c_ for small samples to compare the optimized log-likelihood,

(4) $${\rm{AI}}{{\rm{C}}_{\rm{c}}} =  - 2\log \left( {L(\underline{\hat \Theta}) + {{2K(K + 1)} \over {n - K - 1}}} \right) $$

where *K* is the number of parameters.

AIC scores were compared to the best model $(\Delta {\rm{AI}}{{\rm{C}}_{\rm{c}}} = {\rm{AIC}}-\min {\rm{AIC}})$, and were weighted using Akaike weights

(5) $$w{\rm{AI}}{{\rm{C}}_{\rm{c}}} = {{\rm{e}}^{-0.5\Delta {\rm{AI}}{{\rm{C}}_i}}}\bigg/\left (\sum\nolimits_1^R {{{\rm{e}}^{-0.5\Delta {\rm{AI}}{{\rm{C}}_r}}}}\right ),$$

where *R* is the number of models considered. *w*AIC_c_ provides a weighting indicating the likelihood of a single optimized model compared to all considered models, while penalizing for over-parameterization. Akaike weights were used to calculate evidence ratios relative to the best model (Tables 1 and 2; Tables S1 and S2). See Posada & Buckley (2004) and Symonds & Moussalli (2011) for abbreviated explanations of Akaike weights and evidence ratios.

**Table 1:** Absorptance model comparisons for *Principapillatus hitoyensis* and *Homo sapiens* using maximum likelihood and Akaike’s information criterion corrected for small sample sizes (AIC_c_).

Species or condition	Reference	λ_max1_ (*A*_1_/*A*)	λ_max2_ (*A*_2_/*A*)	λ_max3_ (*A*_3_/*A*)	λ_max4_ (*A*_4_/*A*)	AIC_c_	ΔAIC_c_	*w*AIC_c_	Evidence ratio
	Model								
*Principapillatus hitoyensis*	Beckmann et al. (2015)	484	–	–	–	–	–	–	–
	1, GFKRD	481 (1.0)	–	–	–	55.8	0	0.508	–
	1, SSH^a^	481 (1.0)	–	–	–	54.9	0.863	0.330	1.54
	2, GFKRD^b^	481 (0.70)	481 (0.30)	–	–	53.2	2.54	0.143	3.56
Normal human (scotopic)	Wyszecki & Stiles (2000)	420	497	–	–	–	–	–	–
	2, SSH	421 (0.16)	495 (0.85)	–	–	91.3	0	0.500	–
	2, GFKRD^a^	419 (0.17)	495 (0.83)	–	–	91.1	0.176	0.458	1.09
	3, SSH^b^	407 (0.11)	493 (0.45)	493 (0.45)	–	85.1	6.24	0.02	22.6
Enhanced S-cone human (scotopic)	Jacobson et al. (1990)	420	497	–	–	–	–	–	–
	2, SSH	429 (0.76)	506 (0.24)	–	–	65.6	0	0.587	–
	2, GFKRD^a^	429 (0.75)	506 (0.25)	–	–	64.0	1.62	0.261	2.25
	3, GFKRD^b^	375 (0.27)	432 (0.54)	507 (0.20)	–	62.0	3.79	0.088	6.65

**Notes:**

Photoreceptor arrays were modeled for each species and condition using parameters from Eqs. (1) and (2) (Materials and Methods). *A_i_*/*A*, relative area of photoreceptor in cross-section. SSH, rhodopsin visual pigment template (Stavenga, Smits & Hoenders, 1993). GFRKD, rhodopsin visual pigment template (Govardovskii et al., 2000). Three best-supported models are displayed here for each species or condition. All model comparisons considered are included in Table S1. Evidence ratios were calculated relative to the best model for each species or condition.

a Models with ambiguous *w*AIC_c_ (evidence ratio < 2.0).

b Models with low support relative to the best model (evidence ratio > 2.0).

**Table 2:** Absorptance model comparisons for *Daphnia magna* and *Papilio xuthus* using maximum likelihood and Akaike’s information criterion corrected for small sample sizes (AIC_c_).

Species or condition	Reference	λ_max1_ (*A*_1_/*A*)	λ_max2_ (*A*_2_/*A*)	λ_max3_ (*A*_3_/*A*)	λ_max4_ (*A*_4_/*A*)	λ_max5_ (*A*_5_/*A*)	AIC_c_	ΔAIC_c_	*w*AIC_c_	Evidence ratio
	Model									
*Daphnia magna* (tiered absorptance)	Smith & Macagno (1990)	356	440	521	592	–	–	–	–	–
	4, SSH	362 (0.52)	442 (0.21)	518 (0.12)	587 (0.15)	–	46.2	0	0.979	–
	3, SSH^b^	367 (0.50)	455 (0.22)	560 (0.28)	–	–	38.3	7.96	0.018	53.64
	4, GFKRD^b^	364 (0.50)	437 (0.21)	508 (0.12)	582 (0.17)	–	33.3	12.97	<0.01	656
*Papilio xuthus* (tiered absorptance)	Arikawa, Inokuma & Eguchi (1987)	360	390/400	460	520	600	–	–	–	–
	2, SSH	429 (0.48)	529 (0.52)	–	–	–	34.9	0	0.726	–
	3, SSH^b^	429 (0.56)	505 (0.23)	559 (0.21)	–	–	31.4	3.477	0.128	5.69
	2, GFKRD^b^	422 (0.49)	529 (0.51)	–	–	–	30.5	4.389	0.081	8.98
*Papilio xuthus* (absorbance)	Arikawa, Inokuma & Eguchi (1987)	360	390/400	460	520	600	–	–	–	–
	5, GFKRD	346 (0.10)	381 (0.25)	457 (0.32)	529 (0.20)	586 (0.12)	50.4	0	0.653	–
	3, SSH^b^	371 (0.35)	463 (0.37)	557 (0.28)	–	–	47.8	2.63	0.176	3.71
	4, GFKRD^b^	348 (0.13)	385 (0.26)	465 (0.36)	559 (0.25)	–	46.6	3.83	0.096	6.77

**Notes:**

Tiered photoreceptor arrays were modeled for each species and condition using parameters from Eqs. (1) and (2) (Materials and Methods). *A_i_*/*A*, relative area of photoreceptor in cross-section. SSH, rhodopsin visual pigment template (Stavenga, Smits & Hoenders, 1993). GFRKD, rhodopsin visual pigment template (Govardovskii et al., 2000). Three best-supported models are displayed here for each species or condition. All model comparisons considered are included in Table S2. Evidence ratios were calculated relative to the best model for each species or condition.

a Models with ambiguous *w*AIC_c_ (evidence ratio < 2.0).

b Models with low support relative to the best model (evidence ratio > 2.0).

The above procedure was first used to optimize models to extracellular ERG data for *D. magna*. Beta bands were considered for every possible photoreceptor, an “all subsets” generalized linear model examining the influence of each parameter on *S*(λ) relative to known *S*(λ), comparing among 124 optimized models (Table S4). Generalized linear model results indicated beta bands were uninformative for model selection as they were the least important covariate β, in this case $({{{{\hat \beta }_\beta }} \over {E(yi)}}) < 3.0$, and upon removal led to a reduction in AIC_c_ according to methods outlined in Burnham & Anderson (2002) and Arnold (2010). Models which included beta bands were therefore removed and only models in Tables S1–S3 were included for the formal analysis.

## Results and Discussion

Visual physiologists have long used inferences from thin sections to identify the wavelength of peak absorbance for visual pigments. The reason is the absorbance of visual pigments can be predicted very accurately once the wavelength of peak absorbance, λ_max_, is identified. In practice, this is achieved by excising a portion of the retina, taking sections of the photoreceptors, and measuring the fraction of light which is transmitted or absorbed. Ideally, this is performed on single photoreceptors, using a range of narrow-bandwidth light to infer the wavelength of peak absorbance. Vision researchers found that peak absorbance can be used to normalize the rest of the absorbance curve to create a template curve (Dartnall, 1953). Then, using just the wavelength of peak absorbance, it was found that the rest of the curve can be predicted using mathematical expressions. These nomograms correspond closely to visual pigment that is extracted in solution (Govardovskii et al., 2000). Therefore, the idea of a “universal visual pigment template” is very useful when the wavelength of peak absorbance is known, referred to as “normalized absorption templates”. And because λ_max_ of a visual pigment is primarily determined by the particular opsin amino acids in opsin–chromophore interactions, it is now possible to specify which amino acids determine a specific absorbance profile (Arendt et al., 2004; Porter et al., 2007). However, a normalized absorption template can be misleading when placing the function of a single photoreceptor class in context of other photoreceptors, or the overall spectral sensitivity of the eye. Therefore, absorptance models were used here with the assumption that they are a more realistic approximation for overall sensitivity estimated from extracellular ERGs, and to incorporate multiple layers of filtering.

The first goal of the framework presented here was to find whether overall sensitivity can be used to identify the most likely number of underlying spectral classes of photoreceptors. As can be seen from the fit of each best model to the data (Fig. 1), and from the evidence ratios (Tables 1 and 2), the framework described here is generally able to resolve the number and relative cross-sectional area or frequency of the photoreceptors in the visual systems I have modeled. It is important to note that AIC avoids over-parameterization with the clearest example shown here for velvet worm *Principapillatus hitoyensis*. Though one to five spectral classes were considered (Table 1; Table S1), to add parameters (i.e., more complex models), the likelihood of those models, given the data, must outweigh the penalty imposed by additional parameters. *Principapillatus hitoyensis* sensitivity (Fig. 1A, points) is represented by a single spectral opsin class expressed in its photoreceptors with an estimated λ_max_ of 484 nm, and the best-supported model here was a single receptor GFKRD absorptance model with λ_max_ of 481 nm (Fig. 1A, black curve).

This framework is also able to resolve the presence of more photoreceptors, if the data support them. *Daphnia magna* sensitivity (Fig. 1D) is represented by four spectral photoreceptor classes with a distal UV receptor (Smith & Macagno, 1990), and the best-supported model here was a four receptor SSH absorptance model (Table 2; Table S2). The results strongly support the presence of a UV sensitive photoreceptor in the compound eye of *D. magna*. Though it was poorly supported in comparison to the best model (evidence ratio > 2.0), the second best-supported model for *D. magna* is a three receptor SSH model, rather than a four receptor GFKRD model (Table 2). This finding can be explained by better performance of the SSH template in the UV range, which has been documented (Stavenga, 2010). Future modeling efforts for organisms with UV photoreceptors should expect stronger cumulative performance of absorptance models based on the SSH template.

Results for *Principapillatus hitoyensis* and *D. magna* indicate that this technique resolves a range of opsin-based photoreceptor classes in visual systems. In comparison to more traditional null-hypothesis testing (Table 3), AIC results were similar, with the exception of humans, in which an *F-*test of non-linear regression results would identify three spectral photoreceptor classes. Table 3 also shows how the penalty imposed by AIC for unneeded parameters provides similar results to comparisons of non-linear regression models. Intuitively, this type of multi-model selection should make sense in terms of natural selection, as maintaining photoreceptors is costly, and if they do not match natural spectra, there is an inarguable cost. It should also be emphasized that, to date, *P. hitoyensis* and *D. magna* have not been found to possess specialized optical filtering in their visual systems (Smith & Macagno, 1990; Martin, 1992; Beckmann et al., 2015).

**Table 3:** AIC inferences compared to traditional hypothesis testing which uses an *F*-test to distinguish between two best models of similar fit.

Species or condition	Model	Residual sum of squares (RSS)	*F*-test comparing two models with best fit	*p* Value from *F*-test	Number of parameters (*K*)	Evidence ratio
*Principapillatus hitoyensis*	1, GFKRD	0.031	1.90	0.13	3	–
	2, GFKRD	0.024	–	–	5	3.56
Normal human (scotopic)	2, SSH	0.003	2.75	0.05*	5	–
	3, SSH	0.002	–	–	7	22.6
Enhanced S-cone human (scotopic)	2, SSH	0.012	2.75	0.05*	5	–
	3, GFKRD	0.008	–	–	7	6.65
*D. magna*	4, SSH	0.009	11	<0.001	9	–
	3, SSH	0.031	–	–	7	53.64
*Papilio xuthus* (tiered absorptance)	2, SSH	0.100	2.05	0.10	5	–
	3, SSH	0.076	–	–	7	5.69
*Papilio xuthus* (absorbance)	5, GFKRD	0.006	10.5	<0.001	11	–
	3, SSH	0.034	–	–	7	3.71

**Notes:**

The best model and the closest model with a different number of photoreceptor spectral classes according to AIC are displayed in this order for each species or condition. An *F*-test typically used for comparing non-linear regression models with similar fits was used here to compare two models with lowest residual sum of squares. In cases were *p* < 0.05, the model with more parameters is accepted. Examples which deviated from AIC results are shown with an asterisk (*). This comparison indicates that AIC provides a similar framework to non-linear regression to compare multiple models and can generally eliminate unneeded parameters (in this table, photoreceptor classes and cross-sectional area).

To establish whether this framework can identify the same number and photoreceptor λ_max_ of a visual system when the frequency of the spectral photoreceptor classes is known to differ between individuals, this framework was applied to scotopic human spectral sensitivities. Normal and enhanced S-cone human scotopic sensitivities (Figs. 1B and 1C) are represented by S-cone and rod photoreceptors, with a higher frequency of S cones in patients with enhanced S-cone syndrome (Jacobson et al., 1990; Hood et al., 1995; Haider et al., 2000). Although the full width half-maximum (FWHM) of normal, dark-adapted humans is 20 nm narrower than *Principapillatus hitoyensis* (Fig. 1), the best-supported model using this technique is a two receptor GFKRD absorptance model (Table 1). The narrow bandwidth of normal dark-adapted humans can be explained primarily by the presence of the macula, and illustrates that overlooking absorptive layers which affect spectral sensitivity of underlying photoreceptors leads to erroneous interpretation of the number of spectral photoreceptor classes they possess. As can be seen from Table 1 and Fig. 1, the framework presented here identifies increased frequency of S cones in individuals with enhanced S-cone syndrome, and also identifies two primary spectral photoreceptor classes.

To identify limitations of model over-simplification, I applied this technique to *P. xuthus* sensitivity (Figs. 1E and 1F). Absorptance models (Fig. 1E, dashed lines) illustrate poor results with this technique for *P. xuthus*: as can be seen by the very broad (>100 μm at FWHM) sensitivity of each modeled photoreceptor in the “best” model, self-screening has been over-estimated. *P. xuthus* is known to use specialized filtering pigments in part to sharpen the spectral sensitivity of its receptors (Arikawa, 2003). Opsins are expressed heterogeneously in separate classes of ommatidia leading to regions of their compound eyes differing in spectral sensitivity (Arikawa, Inokuma & Eguchi, 1987; Arikawa & Stavenga, 1997). However, absorbance (Fig. 1F) at cross-section two-thirds from the distal tip of the rhabdom of an ommatidium selects a five spectral photoreceptor GFKRD absorbance model. *P. xuthus* possesses filtering pigments in the peak spectral regions of the photoreceptor classes with the largest deviations identified by this technique (λ_max1_, λ_max2_, and λ_max5_, Table 2). *P. xuthus* is not known to possess filtering pigments in the peak bandwidths of the remaining spectral classes (λ_max3_ and λ_max4_, Table 2) (Wakakuwa, Stavenga & Arikawa, 2007). The comparison of *P. xuthus* absorbance and absorptance results serves to illustrate that multi-model selection must be used judiciously based on what is known for a given visual system. Absorbance results presented here fail to identify the diversity of receptors, and ommatidial spectral classes of organisms where fine-scale spectral discrimination is essential to their visual ecology (Koshitaka et al., 2008). The modeling framework is still useful for incorporating both electrophysiology and histology to compare the effects on overall spectral sensitivity. Deviations from these models can identify the presence of previously unknown spectral filters for an organism, or can provide objective multi-model inference to validate what is known of their visual system.

The examples used until this point are from dark-adapted eyes, and *k*, the peak absorption coefficient in Eq. (2), remained constant. In these examples λ_max_, the wavelength of peak absorbance of each photoreceptor, and *A_i_*/*A*, the relative area or frequency in cross-section of each photoreceptor, were allowed to vary for optimization. However, relative opsin gene expression levels can vary over short time scales (Fuller & Claricoates, 2011), or can change depending on light environment (Fuller, Noa & Strellner, 2010). Therefore, an additional goal of the modeling framework presented here was to use overall sensitivity to map relative opsin expression levels to visual pigment concentration in an organism with well-characterized photoreceptor classes, by allowing *k* to vary. The bluefin killifish, *L. goodei*, was used as two populations found in spring (broad wavelength) and swamp (red-shifted) light environments have been shown to differ in relative opsin expression level for multiple cone photoreceptor classes. The first two rows of Table 4 show the known values of λ_max_, and *A_i_/A* which were entered as constants into this framework, and the final two rows show the expression level of each opsin in proportion to all other opsins which were measured in a real-time PCR study (Fuller et al., 2004).

**Table 4:** Photoreceptor parameters and reported relative opsin expression values for two populations of *L. goodei* used in modeling absorption coefficient *k* for known opsin-based spectral photoreceptor classes.

Species and population	λ_max1_ (*A*_1_/*A*)	Opsin_1_ (exp)	λ_max2_ (*A*_2_/*A*)	Opsin_2_ (exp)	λ_max3_ (*A*_3_/*A*)	Opsin_3_ (exp)	λ_max4_ (*A*_4_/*A*)	Opsin_4_ (exp)	λ_max5_ (*A*_5_/*A*)	Opsin_5_ (exp)
*L. goodei* spring population	359 (0.08)	SWS1 (0.21)	405 (0.31)	SWS2B (0.26)	454 (0.16)	SWS2A (<0.01)	538 (0.25)	RH2-1 (0.27)	572 (0.25)	LWS (0.25)
*L. goodei* swamp population	359 (<0.01)	SWS1 (0.11)	405 (0.16)	SWS2B (0.21)	456 (0.10)	SWS2A (<0.01)	541 (0.32)	RH2-1 (0.33)	573 (0.42)	LWS (0.34)

**Notes:**

Values for λ_max_ and cone frequencies (*A_i_*/*A*) were identified using microspectrophotometry (Fuller et al., 2003). These values were incorporated as constants into model optimization of absorption coefficients below. Relative opsin expression (exp) is in comparison to the sum of all opsins expression is reported from Fuller et al. (2004). Relative expression levels should be compared to Table 5 normalized absorption coefficients.

**Figure 2:**
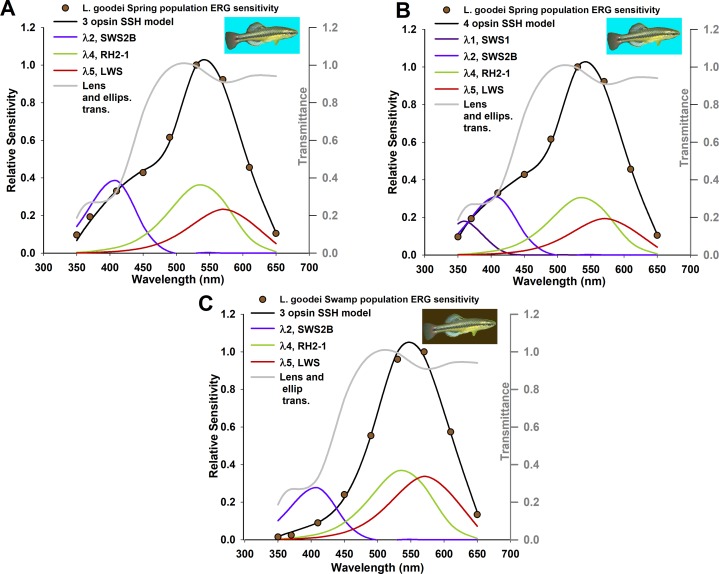
Absorption coefficient models based on known relative opsin expression levels from two populations for the killifish, *Lucania goodei*. Models were fit to relative spectral sensitivity data extracted from published sources (data points). Models were selected using Akaike’s information criterion corrected for small sample sizes (AIC_c_) with the best three models shown in Tables 1 and 2, and all models in Table S3. λ_max_ and *A_i_*/*A* were held constant and not included as parameters.

**Figure 3:**
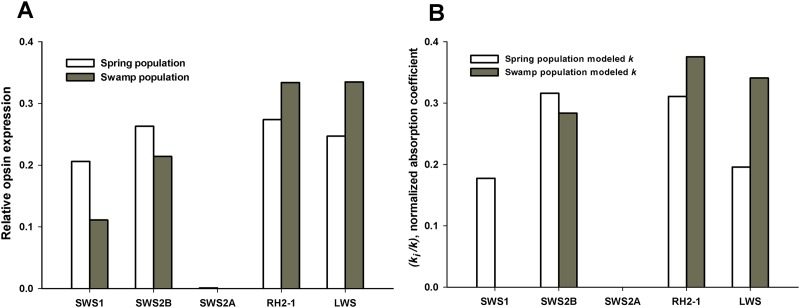
Absorption coefficient values from Table 5 for comparison to relative opsin expression levels from Fuller et al. (2004). Opsin expression was quantified relative to the total opsin expression level.

The alternative hypotheses in this example pertained to the number of photoreceptors that had visual pigments with absorption coefficients *k* < 0.001/μm. The three best models for the spring population are all well supported by the data (evidence ratio < 2.0), indicating that the framework presented here will select the presence of photoreceptors with three or four visual pigments in meaningful concentrations; the model with three visual pigments is supported for the swamp population (Table 5). Though killifish are known to have at least five main spectral cone photoreceptor classes, relative expression levels of class SWS2A reported to date for this species are not found at meaningful expression levels (Table 4) (Fuller et al., 2004). The relative frequency of UV photoreceptors (which express opsin SWS) for swamp populations is less than 0.01 (Table 4), indicating that three visual pigments are likely the main contributors to overall sensitivity. The best SSH models and transmittance through the lens and ellipsosomes are shown in Fig. 2. The optimized values of *k* for each visual pigment were also informative. Though they tended to individually be less than values typically found in vertebrate photoreceptors, the sum of these ranges from 0.0163 in the best four SSH model, to ∼0.0455 in one of three GFKRD models. These are all within the range of *k* typically found in vertebrate photoreceptors (Cronin et al., 2014). These values are informative for two reasons: first, they mean that there are most likely physiological limits to visual pigment concentrations because they are near saturation in photoreceptors, and second, when modeling *k* it is assumed to be at the peak wavelength of each visual pigment, which is not possible at all wavelengths, which has been addressed by Warrant & Nilsson (1998). Further, when *k* is compared to the sum of all *k* values in Fig. 3, it becomes apparent that the main opsin expression results have been reproduced by these optimized models. This indicates that future opsin expression studies, which are often difficult to place in context of either overall sensitivity or behavior (Fuller & Noa, 2010) could use the framework suggested here, and models of overall sensitivity inferred from extracellular ERGS.

**Table 5:** Absorptance model comparisons for two populations of *L. goodei* identify differences in absorption coefficient *k* for known opsin-based spectral photoreceptor classes.

Species and population	Model	SWS1	SWS2B	SWS2A	RH2-1	LWS	AIC_c_	ΔAIC_c_	*w*AIC_c_	Evidence ratio
		*k_1_*	*k_2_*	*k_3_*	*k_4_*	*k_5_*				
		*(k_1_/k)*	*(k_2_/k)*	*(k_3_/k)*	*(k_4_/k)*	*(k_5_/k)*				
*L. goodei* spring population	3, SSH	–	0.0045	–	0.0042	0.0027	37.8	0	0.448	–
		(–)	(0.40)	(–)	(0.37)	(0.24)				
	3, GFKRD^a^	–	0.019	–	0.017	0.0095	37.0	0.819	0.298	1.51
		(–)	(0.42)	(–)	(0.38)	(0.21)				
	4, SSH^a^	0.0030	0.0051	–	0.0050	0.0032	36.7	1.18	0.249	1.80
		(0.18)	(0.32)	(–)	(0.31)	(0.20)				
*L. goodei* swamp population	3, SSH	–	0.0027	–	0.0036	0.0033	37.0	0	0.945	–
		(–)	(0.28)	(–)	(0.38)	(0.34)				
	3, GFKRD^b^	–	0.0077	–	0.0085	0.0074	30.2	6.833	0.031	30.46
		(–)	(0.33)	(–)	(0.36)	(0.31)				
	2, SSH^b^	–	–	–	0.011	0.0092	28.6	8.42	0.014	67.38
		(–)	(–)	(–)	(0.54)	(0.46)				

**Notes:**

Three best-supported models are reported for comparison between absorption coefficients (*k)* normalized by the sum of absorption coefficients (*k_i_*/*k*). All model comparisons considered are included in Table S3. Evidence ratios were calculated relative to the best model for each species or condition.

a Models with ambiguous *w*AIC_c_ (evidence ratio < 2.0).

b Models with low support relative to the best model (evidence ratio > 2.0).

Currently, empirical studies which identify the spectral properties of individual photoreceptor cells or visual pigments are difficult to place in the larger context of the visual system if all the organism’s spectral classes are not identified. The framework I have presented here can be informative for future opsin expression studies and for objectively guiding extracellular or intracellular electroretinography.

## Supplemental Information

10.7717/peerj.3595/supp-1 Supplemental Information 1 Supplementary tables (full model results).

## Additional Information and Declarations

### Data Availability

The following information was supplied regarding data availability: The raw data has been supplied as Supplemental Dataset Files.
