# Supplementary material for: Using electroretinograms and multi-model inference to identify spectral classes of photoreceptors and relative opsin expression levels
Source: PeerJ. 2017 Jul 21;5:e3595. doi: 10.7717/peerj.3595 (PMC5522723; doi:10.7717/peerj.3595)
Supplement: Supplemental Information 1 [file peerj-05-3595-s001.docx]

Table S1.**, related to Table 1. All absorptance model comparisons using maximum likelihood and Akaike’s Information Criterion corrected for small sample sizes (AIC_c_) considered for *Principapillatus hitoyensis* and *Homo sapiens.* Tiered photoreceptor arrays were modeled for each species or condition using parameters from Equations 1 and 2 (Materials and Methods). A_i_/A, relative area or frequency of photoreceptor in cross-section. SSH, rhodopsin visual pigment template** (Stavenga et al., 1993)**. GFRKD, rhodopsin visual pigment template** (Govardovskii et al., 2000)**. Three best supported models** (>0.02 *w*AIC_c_) **are displayed in main text Table 1. Evidence ratios were calculated relative to the best model for each species and sex. Models with ambiguous *w*AIC_c_ (evidence ratio < 2.0) are indicated by (^a^). Models with low support relative to the best model (evidence ratio > 2.0) are indicated by (^b^).**

| **Species or Condition** | **(Reference) Model** |  | **λmax_1_** | **λmax_2_** | **λmax_3_** | **λmax_4_** | **λmax_5_** |  | **A_1_/A** | **A_2_/A** | **A_3_/A** | **A_4_/A** | **A_5_/A** |  | **AIC_c_** | **ΔAIC_c_** | ***w*AIC_c_** | **Evidence Ratio** |
| --- | --- | --- | --- | --- | --- | --- | --- | --- | --- | --- | --- | --- | --- | --- | --- | --- | --- | --- |
| *P.hitoyensis* | (Beckmann et al., 2015) |  | 484 | - | - | - | - |  | - | - | - | - | - |  | - | - | 0.508 | - |
|  | 1, GFKRD^a^ |  | 481 | - | - | - | - |  | 1.0 | - | - | - | - |  | 55.6 | 0 | 0.33 | - |
|  | 1, SSH ^a^ |  | 481 | - | - | - | - |  | 1.0 | - | - | - | - |  | 54.9 | 0.86 | 0.143 | 1.54 |
|  | 2, GFKRD^b^ |  | 481 | 481 | - | - | - |  | 0.70 | 0.30 | - | - | - |  | 53.2 | 2.54 | 0.0159 | 3.56 |
|  | 2, SSH^b^ |  | 481 | 481 | - | - | - |  | 0.18 | 0.82 | - | - | - |  | 48.8 | 6.92 | <0.01 | 31.9 |
|  | 3, GFKRD^b^ |  | 480 | 480 | 630 | - | - |  | 0.54 | 0.42 | 0.03 | - | - |  | 44.9 | 10.9 | <0.01 | 231.3 |
|  | 3, SSH^b^ |  | 481 | 481 | 630 | - | - |  | 0.44 | 0.52 | 0.04 | - | - |  | 39.6 | 16.2 | <0.01 | 3.28x10^3^ |
|  | 4, GFKRD^b^ |  | 330 | 480 | 480 | 630 | - |  | 0.04 | 0.53 | 0.40 | 0.03 | - |  | 31.1 | 24.6 | <0.01 | 2.23x10^5^ |
|  | 4, SSH^b^ |  | 481 | 481 | 481 | 630 | - |  | 0.04 | 0.60 | 0.32 | 0.04 | - |  | 24.3 | 31.5 | <0.01 | 6.89x10^6^ |
|  | 5, GFKRD^b^ |  | 330 | 481 | 480 | 480 | 630 |  | 0.04 | 0.66 | 0.14 | 0.13 | 0.03 |  | 5.63 | 50.1 | <0.01 | 7.68x10^10^ |
|  | 5, SSH^b^ |  | 481 | 481 | 481 | 481 | 630 |  | 0.10 | 0.04 | 0.78 | 0.04 | 0.04 |  | 1.23 | 57.0 | <0.01 | 2.37x10^12^ |
| Normal Human (scotopic) | (Wyszecki and Stiles, 2000) |  | 420 | 497 |  |  |  |  |  |  |  |  |  |  |  |  |  |  |
|  | 2, SSH ^a^ |  | 421 | 495 | - | - | - |  | 0.16 | 0.86 | - | - | - |  | 91.3 | 0 | 0.500 | - |
|  | 2, GFKRD^a^ |  | 419 | 495 | - | - | - |  | 0.17 | 0.83 | - | - | - |  | 91.1 | 0.176 | 0.458 | 7.402 |
|  | 3, SSH^b^ |  | 407 | 493 | 493 | - | - |  | 0.11 | 0.44 | 0.44 | - | - |  | 85.1 | 6.24 | 0.0221 | 1.09 |
|  | 3, GFKRD^b^ |  | 404 | 493 | 493 | - | - |  | 0.14 | 0.43 | 0.43 | - | - |  | 84.8 | 6.50 | 0.0194 | 22.59 |
|  | 4, SSH^b^ |  | 387 | 491 | 491 | 491 | - |  | 0.11 | 0.26 | 0.31 | 0.33 | - |  | 71.5 | 19.8 | <0.01 | 25.75 |
|  | 4, GFKRD^b^ |  | 392 | 491 | 491 | 491 | - |  | 0.13 | 0.33 | 0.21 | 0.33 | - |  | 71.2 | 20.1 | <0.01 | 2.00 x10^4^ |
|  | 1, SSH^b^ |  | 493 | - | - | - | - |  | 1.0 | - | - | - | - |  | 70.0 | 21.3 | <0.01 | 2.31 x10^4^ |
|  | 1, GFKRD^b^ |  | 493 | - | - | - | - |  | 1.0 | - | - | - | - |  | 67.9 | 23.4 | <0.01 | 4.19 x10^4^ |
|  | 5, SSH^b^ |  | 387 | 491 | 491 | 491 | 491 |  | 0.11 | 0.26 | 0.28 | 0.33 | 0.03 |  | 46.1 | 45.3 | <0.01 | 1.22 x10^5^ |
|  | 5, GFKRD^b^ |  | 392 | 491 | 491 | 491 | 491 |  | 0.13 | 0.25 | 0.27 | 0.33 | 0.02 |  | 45.7 | 45.6 | <0.01 | 6.88 x10^9^ |
| Enchanced S-cone Human (scotopic) | (Jacobson et al., 1990) |  | 420 | 497 |  |  |  |  |  |  |  |  |  |  |  |  |  |  |
|  | 2, SSH ^a^ |  | 429 | 506 | - | - | - |  | 0.76 | 0.24 | - | - | - |  | 65.6 | 0 | 0.587 | - |
|  | 2, GFKRD^a^ |  | 429 | 504 | - | - | - |  | 0.75 | 0.25 | - | - | - |  | 64.0 | 1.62 | 0.261 | 2.25 |
|  | 3, GFKRD^b^ |  | 375 | 433 | 507 | - | - |  | 0.27 | 0.54 | 0.20 | - | - |  | 61.8 | 3.79 | 0.088 | 6.65 |
|  | 3, SSH^b^ |  | 383 | 436 | 509 | - | - |  | 0.28 | 0.53 | 0.20 | - | - |  | -61.1 | 4.48 | 0.0626 | 9.38 |
|  | 4, GFKRD^b^ |  | 388 | 432 | 497 | 544 | - |  | 0.22 | 0.55 | 0.20 | 0.03 | - |  | 51.0 | 14.6 | <0.01 | 1.49 x10^3^ |
|  | 4, SSH ^b^ |  | 368 | 430. | 498 | 541 | - |  | 0.29 | 0.52 | 0.16 | 0.03 | - |  | 49.1 | 16.4 | <0.01 | 3.72 x10^3^ |
|  | 1, SSH^b^ |  | 427 | 427 | 427 | 499 | 567 |  | 0.26 | 0.26 | 0.26 | 0.20 | 0.01 |  | 28.6 | 37.0 | <0.01 | 1.06 x10^8^ |
|  | 1, GFKRD^b^ |  | 367 | 429 | 429 | 450 | 558 |  | 0.27 | 0.28 | 0.28 | 0.16 | 0.01 |  | 25.4 | 40.2 | <0.01 | 5.35 x10^8^ |
|  | 5, SSH^b^ |  | 453 | - | - | - | - |  | 1.0 | - | - | - | - |  | 5.05 | 70.6 | <0.01 | 2.16x10^15^ |
|  | 5, GFKRD^b^ |  | 454 | - | - | - | - |  | 1.0 | - | - | - | - |  | 5.74 | 71.3 | <0.01 | 3.06x10^15^ |

Table S2.**, related to Table 2. All absorptance model comparisons using maximum likelihood and Akaike’s Information Criterion corrected for small sample sizes (AIC_c_) considered for *Daphnia magna* and *Papilio xuthus* and absorbance models for *P. xuthus.* Tiered photoreceptor arrays were modeled for each species and sex using parameters from Equations 1 and 2 (Materials and Methods). A_i_/A, relative area of photoreceptor in cross-section. SSH, rhodopsin visual pigment template** (Stavenga et al., 1993)**. GFRKD, rhodopsin visual pigment template** (Govardovskii et al., 2000)**. Three best supported models** (>0.02 *w*AIC_c_) **are displayed in main text Table 2. Evidence ratios were calculated relative to the best model for each species and sex. Models with ambiguous *w*AIC_c_ (evidence ratio < 2.0) are indicated by (^a^). Models with low support relative to the best model (evidence ratio > 2.0) are indicated by (^b^).**

| **Species or Condition** | **(Reference) Model** |  | **λmax_1_** | **λmax_2_** | **λmax_3_** | **λmax_4_** | **λmax_5_** |  | **A_1_/A** | **A_2_/A** | **A_3_/A** | **A_4_/A** | **A_5_/A** |  | **AIC_c_** | **ΔAIC_c_** | ***w*AIC_c_** | **Evidence Ratio** |
| --- | --- | --- | --- | --- | --- | --- | --- | --- | --- | --- | --- | --- | --- | --- | --- | --- | --- | --- |
| *D. magna* (Tiered absorptance) | (Smith and Macagno, 1990) |  | 356 | 440 | 521 | 592 | - |  | - | - | - | - | - |  | - | - | - | - |
|  | 4, SSH^a^ |  | 362 | 442 | 518 | 587 | - |  | 0.52 | 0.21 | 0.12 | 0.15 | - |  | 46.2 | 0 | 0.98 | - |
|  | 3, SSH^b^ |  | 367 | 455 | 560 | - | - |  | 0.50 | 0.22 | 0.28 | - | - |  | 38.3 | 7.96 | 0.02 | 53.6 |
|  | 4, GFKRD^b^ |  | 364 | 437 | 509 | 582 | - |  | 0.50 | 0.21 | 0.12 | 0.16 | - |  | 33.3 | 13.0 | <0.01 | 656.0 |
|  | 3, GFKRD^b^ |  | 370 | 453 | 558 | - | - |  | 0.49 | 0.23 | 0.28 | - | - |  | 31.8 | 14.4 | <0.01 | 1.35 x10^3^ |
|  | 5, GFKRD^b^ |  | 353 | 381 | 446 | 523 | 591 |  | 0.35 | 0.16 | 0.23 | 0.13 | 0.14 |  | 27.7 | 18.5 | <0.01 | 1.05 x10^4^ |
|  | 5, SSH^b^ |  | 358 | 395 | 450 | 528 | 594 |  | 0.48 | 0.07 | 0.21 | 0.12 | 0.12 |  | 26.0 | 20.2 | <0.01 | 2.48 x10^4^ |
|  | 2, SSH^b^ |  | 378 | 492 | - | - | - |  | 0.70 | 0.30 | - | - | - |  | 4.08 | 50.3 | <0.01 | 8.45x10^10^ |
|  | 2, GFKRD^b^ |  | 380 | 488 | - | - | - |  | 0.69 | 0.31 | - | - | - |  | 5.71 | 52.0 | <0.01 | 1.91x10^11^ |
|  | 1, SSH^b^ |  | 440 | - | - | - | - |  | 1.0 | - | - | - | - |  | 30.0 | 76.2 | <0.01 | 3.52x10^16^ |
|  | 1, GFKRD^b^ |  | 440 | - | - | - | - |  | 1.0 | - | - | - | - |  | 31.1 | 77.3 | <0.01 | 6.11x10^16^ |
| *P. xuthus* (Tiered absorptance) | (Arikawa et al., 1987) |  | 360 | 390  /400 | 460 | 520 | 600 |  | - | - | - | - | - |  | - | - | - | - |
|  | 2, SSH^a^ |  | 429 | 529 | - | - | - |  | 0.48 | 0.52 | - | - | - |  | 34.9 | 0 | 0.726 | - |
|  | 3, SSH^b^ |  | 429 | 505 | 559 | - | - |  | 0.56 | 0.23 | 0.21 | - | - |  | 31.4 | 3.48 | 0.128 | 5.69 |
|  | 2, GFKRD^b^ |  | 422 | 529 | - | - | - |  | 0.49 | 0.51 | - | - | - |  | 30.5 | 4.39 | 0.0808 | 8.98 |
|  | 3, GFKRD^b^ |  | 418 | 491 | 548 | - | - |  | 0.50 | 0.23 | 0.28 | - | - |  | 30.0 | 4.81 | 0.0655 | 11.08 |
|  | 4, SSH^b^ |  | 429 | 498 | 542 | 604 | - |  | 0.60 | 0.21 | 0.15 | 0.04 | - |  | 19.3 | 15.6 | <0.01 | 2.42 x10^3^ |
|  | 4, GFKRD^b^ |  | 418 | 4823 | 532 | 591 | - |  | 0.55 | 0.22 | 0.18 | 0.06 | - |  | 18.3 | 16.6 | <0.01 | 4.03 x10^3^ |
|  | 5, GFKRD^b^ |  | 425 | 490 | 536 | 536 | 600 |  | 0.57 | 0.21 | 0.12 | 0.06 | 0.05 |  | 2.00 | 32.8 | <0.01 | 1.35 x10^7^ |
|  | 5, SSH^b^ |  | 436 | 506 | 547 | 547 | 616 |  | 0.63 | 0.21 | 0.10 | 0.03 | 0.03 |  | 0.27 | 34.6 | <0.01 | 3.24 x10^7^ |
|  | 1, SSH^b^ |  | 479 | - | - | - | - |  | 1.0 | - | - | - | - |  | 5.05 | 39.9 | <0.01 | 4.63 x10^8^ |
|  | 1, GFKRD^b^ |  | 471 | - | - | - | - |  | 1.0 | - | - | - | - |  | 13.4 | 48.2 | <0.01 | 2.94x10^10^ |
| *P. xuthus* (Absorbance) | (Arikawa et al., 1987) |  | 360 | 390  /400 | 460 | 520 | 600 |  | - | - | - | - | - |  | - | - | - | - |
|  | 5, GFKRD^a^ |  | 346 | 381 | 457 | 529 | 586 |  | 0.10 | 0.25 | 0.32 | 0.20 | 0.12 |  | 50.4 | 0 | 0.653 | - |
|  | 3, SSH^b^ |  | 371 | 462 | 557 | - | - |  | 0.35 | 0.37 | 0.28 | - | - |  | 47.8 | 2.62 | 0.176 | 3.71 |
|  | 4, GFKRD^b^ |  | 348 | 385 | 465 | 559 | - |  | 0.12 | 0.26 | 0.36 | 0.25 | - |  | 46.6 | 3.83 | 0.0963 | 6.77 |
|  | 4, SSH^b^ |  | 353 | 387 | 467 | 560 | - |  | 0.16 | 0.22 | 0.37 | 0.26 | - |  | 45.7 | 4.72 | 0.0617 | 10.6 |
|  | 5, SSH^b^ |  | 349 | 380 | 461 | 538 | 595 |  | 0.11 | 0.24 | 0.34 | 0.22 | 0.10 |  | 42.0 | 8.41 | <0.01 | 67.2 |
|  | 3, GFKRD^b^ |  | 373 | 459 | 555 | - | - |  | 0.35 | 0.37 | 0.28 | - | - |  | 40.1 | 10.4 | <0.01 | 174.0 |
|  | 2, SSH^b^ |  | 392 | 512 | - | - | - |  | 0.48 | 0.52 | - | - | - |  | 3.00 | 53.4 | <0.01 | 3.93x10^11^ |
|  | 2, GFKRD^b^ |  | 393 | 509 | - | - | - |  | 0.49 | 0.51 | - | - | - |  | 5.04 | 55.4 | <0.01 | 1.09x10^11^ |
|  | 1, SSH^b^ |  | 480 | - | - | - | - |  | 1.0 | - | - | - | - |  | 33.0 | 83.4 | <0.01 | 1.30x10^18^ |
|  | 1, GFKRD^b^ |  | 480 | - | - | - | - |  | 1.0 | - | - | - | - |  | 34.4 | 84.7 | <0.01 | 2.52x10^18^ |

Table S3.**, related to Table 5. All absorptance model comparisons using maximum likelihood and Akaike’s Information Criterion corrected for small sample sizes (AIC_c_) considered two populations of *L goodei* identify differences in absorption coefficient *k* for known opsin-based spectral photoreceptor classes*.*Tiered photoreceptor arrays were modeled for each species and sex using parameters from Equations 1 and 2 (Materials and Methods).** Absorption coefficients (*k)* normalized by the sum of absorption coefficients *(k_i_/k).* **GFRKD, rhodopsin visual pigment template** (Govardovskii et al., 2000)**. Three best supported models** (>0.01 *w*AIC_c_) **are displayed in main text Table 2. Evidence ratios were calculated relative to the best model for each species and sex. Models with ambiguous *w*AIC_c_ (evidence ratio < 2.0) are indicated by (^a^). Models with low support relative to the best model (evidence ratio > 2.0) are indicated by (^b^).**

| **Species or Condition** | **Model** |  |  | ***SWS1***  ***k_1_***  ***(k_1_/k)*** | ***SWS2B***  ***k_2_***  ***(k_2_/k)*** | ***SWS2A***  ***k_3_***  ***(k_3_/k)*** | ***RH2-1***  ***k_4_***  ***(k_4_/k)*** | ***LWS***  ***k_5_***  ***(k_5_/k)*** |  | **AIC_c_** | **ΔAIC_c_** | ***w*AIC_c_** | **Evidence Ratio** |
| --- | --- | --- | --- | --- | --- | --- | --- | --- | --- | --- | --- | --- | --- |
| *L. goodei* Spring population | 3,SSH^a^ |  |  | -  (-) | 0.0045  (0.40) | -  (-) | 0.0042 (0.37) | 0.0027  (0.24) |  | 37.8 | 0 | 0.448 | - |
|  | 3,GFKRD^a^ |  |  | -  (-) | 0.019 (0.42) | -  (-) | 0.017  (0.38) | 0.0095  (0.21) |  | 37.0 | 0.819 | 0.298 | 1.51 |
|  | 4,SSH^a^ |  |  | 0.0030  (0.18) | 0.0051 (0.32) | -  (-) | 0.0050  (0.31) | 0.0032 (0.20) |  | 36.7 | 1.18 | 0.249 | 1.80 |
|  | 4, GFKRD^b^ |  |  | 0.0035 (0.071) | 0.019  (0.38) | -  (-) | 0.017 (0.35) | 0.0096  (0.19) |  | 28.9 | 8.91 | <0.01 | 86.2 |
|  | 5, GFKRD^b^ |  |  | 0.0022 (0.16) | 0.004 (0.30) | 0.001  (0.07) | 0.0038  (0.28) | 0.0025  (0.19) |  | 21.6 | 16.2 | <0.01 | 3.35x10^3^ |
|  | 5, SSH^b^ |  |  | 0.0064 (0.24) | 0.0071 (0.26) | 0.001  (0.037 | 0.0076  (0.28) | 0.0025 (0.18) |  | 19.2 | 18.7 | <0.01 | 1.13 x10^4^ |
|  | 2, GFKRD^b^ |  |  | -  (-) | 0.057 (0.37) | -  (-) | 0.10  (0.633194157) | 0.0049  (-) |  | 12.7 | 25.1 | <0.01 | 2.88 x10^5^ |
|  | 2, SSH^b^ |  |  | -  (-) | 0.048 (0.33) | -  (-) | 0.097  (0.67017793) | -  (-) |  | 11.8 | 26.0 | <0.01 | 4.50 x10^6^ |
|  | 1, SSH^b^ |  |  | 1  (-) | -  (-) | -  (-) | 0.15  (1) | -  (-) |  | 6.40 | 31.4 | <0.01 | 6.70 x10^6^ |
|  | 1, GFKRD^b^ |  |  | 1  (-) | -  (-) | -  (-) | 0.15  (1) | -  (-) |  | 4.92 | 32.9 | <0.01 | 1.40 x10^7^ |
|  |  |  |  |  |  |  |  |  |  |  |  |  |  |
| *L. goodei* Swamp population | 3,SSH^b^ |  |  | -  (-) | 0.0027 (0.28) | -  (-) | 0.0036 (0.38) | 0.0033  (0.34) |  | 37.0 | 0 | 0.945 | - |
|  | 3,GFKRD^b^ |  |  | -  (-) | 0.0077 (0.33) | -  (-) | 0.0085 (0.36) | 0.0074 (0.31) |  | 30.2 | 6.833 | 0.031 | 30.46 |
|  | 2,SSH^b^ |  |  | -  (-) | -  (-) | -  (-) | 0.011 (0.54) | 0.0092 (0.46) |  | 28.6 | 8.42 | 0.014 | 67.38 |
|  | 4,SSH^b^ |  |  | 0.001  (0.09) | 0.0028  (0.26) | -  (-) | 0.0037  (0.34) | 0.0033  (0.31) |  | 27.9 | 9.09 | 0.010 | 94.34 |
|  | 4, GFKRD^b^ |  |  | 0.001  (0.04) | 0.0077  (0.31) | -  (-) | 0.008  (0.35) | 0.0074  (0.30) |  | 21.1 | 15.9 | <0.01 | 2.77 x10^3^ |
|  | 5,SSH^b^ |  |  | 0.001  (0.08) | 0.0023  (0.19) | 0.001  (0.08) | 0.004  (0.34) | 0.0039  (0.31) |  | 11.8 | 25.2 | <0.01 | 2.90 x10^5^ |
|  | 5, GFKRD^b^ |  |  | 0.001  (0.13) | 0.0016  (0.20) | 0.001  (0.13) | 0.002  (0.28) | 0.0021  (0.27) |  | 11.3 | 25.7 | <0.01 | 3.78 x10^5^ |
|  | 1, GFKRD^b^ |  |  | -  (-) | -  (-) | -  (-) | 0.13  (1) | -  (-) |  | 8.07 | 28.9 | <0.01 | 1.91 x10^6^ |
|  | 1,SSH^b^ |  |  | -  (-) | -  (-) | -  (-) | 0.13  (1) | -  (-) |  | 7.97 | 29.0 | <0.01 | 2.00 x10^6^ |
|  | 2, GFKRD^b^ |  |  | -  (-) | 0.012  (0.08) | -  (-) | 0.13  (0.92) | -  (-) |  | 3.83 | 33.2 | <0.01 | 1.59 x10^7^ |

Table S4. All subsets generalized linear model examining influence of each variable on $\boldsymbol{S(\lambda)}$**relative to known values of**$\boldsymbol{S(\lambda)}$ of *Daphnia magna* ${\sum\left( \left( \boldsymbol{S(\lambda)}_{\boldsymbol{model}}\boldsymbol{-}\boldsymbol{S(\lambda)}_{\boldsymbol{known}} \right)^{\boldsymbol{2}} \right)\boldsymbol{.}}$

This was carried out for *D. magna* only as it possesses more spectral photoreceptor classes underlying its dark-acclimated $\boldsymbol{S(\lambda)}$ **than either *Principapillatus hitoyensis* or *Homo sapiens.***

| **Variable** | **Regression coefficient**  $\beta_{\beta}$ | **Std error**  $E(yi)$ | $\frac{\beta_{\beta}}{E(yi)}$ |
| --- | --- | --- | --- |
| λmax_i_ | -1.816 | 0.026 | 55.03 |
| β band on λmax_1_ | -0.06 | 0.046 | 1.04 |
| β band on λmax_2_ | -0.096 | 0.046 | 1.66 |
| β band on λmax_3_ | -0.126 | 0.048 | 2.1 |
| β band on λmax_4_ | 0.053 | 0.051 | 0.828 |
| β band on λmax_5_ | 0.063 | 0.056 | 0.851 |
|  |  |  |  |
